# Effectiveness of Medical Treatment on Survivability in Canine Cushing’s Syndrome: A Systematic Review and Meta-Analysis

**DOI:** 10.3390/ani15202954

**Published:** 2025-10-12

**Authors:** Sophia Shanlly, Jordan Slessor, Wenting Yan, Jessica J. D. Thorlakson, Heather L. Bruce, Richard R. E. Uwiera

**Affiliations:** 1 Department of Agricultural, Food & Nutritional Science, University of Alberta, Edmonton, AB T6G 2R3, Canada; sshanlly@ualberta.ca; 2 Department of Mathematical & Statistical Sciences, University of Alberta, Edmonton, AB T6G 2R3, Canada; jaslesso@ualberta.ca; 3 Faculty of Nursing, University of Alberta, Edmonton, AB T6G 2R3, Canada; wyan4@ualberta.ca; 4 Cameron Science and Engineering Library, University of Alberta, Edmonton, AB T6G 2R3, Canada; jthorlak@ualberta.ca; 5 Faculty of Agriculture, Dalhousie University, Bible Hill, NS B2N 5E3, Canada; heather.bruce@dal.ca

**Keywords:** canine, Cushing’s syndrome, medical treatment, pharmacotherapy, trilostane, mitotane

## Abstract

**Simple Summary:**

Cushing’s syndrome is a common endocrine disorder in dogs characterized by prolonged exposure to excessive cortisol. Several pharmacological agents, including trilostane, mitotane, ketoconazole, cabergoline, selegiline, and aminoglutethimide, are used to manage the condition, but their impact on survival outcomes remains uncertain. This systematic review and meta-analysis evaluated the effects of all these treatments on survival time in dogs with naturally occurring Cushing’s syndrome. Five studies involving 295 dogs met the inclusion criteria. The results showed that trilostane may provide a long-term survival benefit compared to mitotane, with an 11% higher survival rate at 36 months (risk difference: −0.11; 95% CI: −0.15 to −0.06). Comparatively, the other pharmacological agents showed no treatment effect. These findings support trilostane as a more effective treatment option for Cushing’s syndrome in dogs compared to mitotane and underscores the need for prospective trials with standardized outcomes to strengthen the evidence base for veterinary clinical care.

**Abstract:**

Cushing’s syndrome is one of the most common endocrine disorders in dogs and is typically managed with long-term medical treatment. Several pharmacological agents are available: trilostane, mitotane, ketoconazole, cabergoline, selegiline, and aminoglutethimide, but their comparative effects on survival remain unclear. This systematic review and meta-analysis compared the impact of these agents on survival outcomes in dogs with naturally occurring diseases. A comprehensive search of MEDLINE, Embase, Web of Science, Academic Search Complete, and the Cochrane Library was conducted between 1 September 2024 to 3 January 2025. Eligible studies included dogs diagnosed with Cushing’s syndrome that reported survival outcomes for at least one of the specified treatments. Five studies (n = 295 dogs) met the inclusion criteria, with trilostane and mitotane providing sufficient data for meta-analysis. Pooled mean difference in survival time across four studies was 85.1 days (95% CI: −255.9 to 85.7, *p* = 0.21) with substantial heterogeneity (I^2^ = 89%), indicating no statistically significant difference between the drugs. In contrast, pooled survival rates at fixed intervals favored trilostane, with an 11% higher survival at 36 months (*p* = 0.005) and no heterogeneity observed (I^2^ = 0%). These findings suggest trilostane may offer long-term survival benefits over mitotane.

## 1. Introduction

Cushing’s syndrome (CS), or hypercortisolism, is a chronic endocrine disorder in dogs characterized by sustained overproduction of glucocorticoids. This condition may arise from prolonged administration of corticosteroid medications or endogenous overproduction of cortisol due to dysregulation within the hypothalamic–pituitary–adrenal axis or adrenal cortical neoplasia [1]. Naturally occurring CS is one of the most commonly diagnosed endocrine disorders in dogs, with an estimated incidence of 1–2 cases per 1000 dogs annually [2].

Approximately 80–85% of naturally occurring cases are caused by adrenocorticotropic hormone (ACTH)-secreting pituitary adenomas, referred to as pituitary-dependent hypercortisolism (PDH) [2,3,4]. In PDH, excessive ACTH secretion stimulates both adrenal glands, which leads to bilateral adrenal hyperplasia and elevated cortisol levels [5]. Clinical signs typically develop gradually and often include polyuria, polydipsia, polyphagia, abdominal enlargement due to fat redistribution, hepatomegaly, skin thinning, alopecia, and poor wound healing [6]. Although PDH can cause significant morbidity, long-term survival is generally favorable with appropriate medical management.

The remaining 15–20% are most often caused by cortisol-secreting adrenal tumors, typically adrenocortical adenomas or carcinomas, referred to as adrenal-dependent hypercortisolism (ADH) [4]. In ADH, cortisol overproduction originates from a single adrenal gland, while the contralateral gland often becomes atrophic [5]. Clinical signs may develop more acutely and can include severe polyuria, polydipsia, polyphagia, or abdominal enlargement, and occasionally with a palpable adrenal mass [5,6]. Prognosis depends on tumor type and tumor extent. Treatment involves surgical excision of the tumor, which can be curative for adenomas, whereas adrenal carcinomas carry a higher risk of recurrence, metastasis, and reduced long-term survival [6]. Other etiologies, such as ectopic ACTH secretion from non-pituitary tissues or food-dependent hypercortisolism, are exceedingly rare in dogs [5].

Management of CS generally involves pharmacological agents that suppress cortisol production and slow disease progression, including trilostane, mitotane, ketoconazole, cabergoline, selegiline, and aminoglutethimide [7,8,9,10,11,12,13,14]. These treatments differ in their mechanisms of action, efficacy, and safety profiles, and selection is guided by disease severity, comorbidities, clinician experience, and individual patient response [15,16]. Despite the availability of multiple pharmacological treatment options, their comparative effectiveness remains uncertain. Differences in study design, treatment protocols, and outcome measures have limited the ability to draw definitive conclusions. No systematic review and meta-analysis have comprehensively evaluated the impact of these agents on survival in affected dogs.

The objective of this systematic review and meta-analysis is to evaluate and compare the effects of six commonly used pharmacological agents, including trilostane, mitotane, ketoconazole, cabergoline, selegiline, and aminoglutethimide, on survival time with naturally occurring CS in dogs. This systematic review and meta-analysis aim to guide evidence-based treatment selection and identify areas where further research is warranted to improve long-term survival in affected dogs.

## 2. Materials and Methods

### 2.1. Search Strategy

This systematic review and meta-analysis followed the Preferred Reporting Items for Systematic Reviews and Meta-Analyses (PRISMA) 2020 guidelines and the Cochrane Review Methods. Prior to data extraction and analysis, the protocol was registered with the Open Science Framework on 4 January 2025 (doi.org/10.17605/OS F.IO/5XW2C). A comprehensive search was performed in MEDLINE (1946-present via Ovid), Embase (1974-present via Ovid), Web of Science-All Databases (Clarivate Analytics), Academic Search Complete (EBSCO), and the Cochrane Library (Wiley Online Library) from 1 September 2024, to 3 January 2025, to identify all relevant studies evaluating pharmacological treatments for Cushing’s syndrome in dogs. The following search terms were applied: (exp Dogs/ OR canine* OR dog OR dogs OR doggy OR puppy OR puppies OR mongrel* OR hound OR hounds OR terrier* OR spaniel* OR retriever* OR mastiff* OR pinscher* OR collie* OR poodle* OR dachshund* OR corgi* OR shepherd* OR sheepdog* OR beagle* OR coonhound* OR bloodhound* OR borzoi* OR english foxhound* OR greyhound* OR harrier* OR irish wolfhound* OR otterhound* OR rhodesian ridgeback* OR scottish deerhound* OR pooch* OR mutt OR mutts OR bitch*) AND (exp Cushing Syndrome/OR exp Pituitary ACTH Hypersecretion/OR exp ACTH-Secreting Pituitary Adenoma/OR exp Adrenal Gland Neoplasms/OR exp Adrenocorticotropic Hormone/OR exp Adrenocortical Hyperfunction/OR exp Hydrocortisone/OR cushing* OR adrenocorticotropic hormone* OR ACTH* OR ((corticotrop* OR pituitary OR adrenal) ADJ2 (adenoma* OR tumo?r*)) OR hyperadrenocortic* OR hypercortisol* OR hydrocortisone*) AND (exp Drug Therapy/OR exp Mitotane/OR exp Selegiline/OR exp Ketoconazole/OR exp Cabergoline/OR exp Aminoglutethimide/OR pharmacotherap* OR mitotan* OR “o,p-DDD” OR Lysodren OR selegilin* OR l-deprenyl OR Anipryl OR Eldepryl OR Carbex OR Zelepar OR Zelapar OR trilostan* OR Vetoryl OR Desopan OR Modrastan* OR Modrenal OR ketoconazol* OR “R 41400” OR “R41400” OR “R41 40” OR Nizoral OR cabergolin* OR Galastop OR “FCE 21336” OR Caberlin* OR Cabaser OR Cabaseril OR Dostinex OR aminoglutethimid* OR Elipten OR Cytadren OR Orimeten) AND ((medic* OR pharmacolog* OR drug*) ADJ2 (treat* OR therap* OR management* OR intervention* OR efficac* OR effectiv* OR safety OR trial* OR procedure*)). Exploded Medical Subject Headings (MeSH) were used where appropriate, and all identified keywords and index terms were adapted for each individual database to ensure a comprehensive search strategy. No restrictions were applied regarding language, publication date, or publication status, allowing the inclusion of all potentially relevant studies. Reference lists of included articles were manually screened, and Google Scholar was searched to capture any additional eligible publications not indexed in the selected databases. Detailed search strategies for each database are presented in Supplementary Materials, Tables S1–S5. All search results were imported into Covidence for systematic management, and duplicate records were identified and then removed prior to screening to streamline the review process.

### 2.2. Eligibility Criteria

The primary literature included in this systematic review met predefined inclusion and exclusion criteria. Eligible studies involved dogs diagnosed with CS and reported the dosage and duration of at least one pharmacological treatment, and these included mitotane, trilostane, selegiline, ketoconazole, cabergoline, or aminoglutethimide. Each study was required to include a comparator pharmacological agent and to report outcome measures related to survival time or survival rate. Only randomized controlled trials and cohort studies were considered eligible for the analysis. Studies that were in vitro, case reports, or lacked treatment comparisons or relevant survival data were excluded. Additionally, reviews, editorials, letters, and non-peer-reviewed publications were not considered eligible for this study. No minimum follow-up period after CS diagnosis was required, and diagnostic accuracy of the tests used to confirm CS was not part of the eligibility criteria. In the end, all studies that met the inclusion criteria were non-randomized cohort studies.

### 2.3. Data Extraction

Screening and study selection were conducted using Covidence (Veritas Health Innovation, Melbourne, Australia). Two reviewers (S.S. and W.Y.) independently screened titles, abstracts, and full-text articles according to predefined eligibility criteria. A third reviewer (R.R.E.U.) verified the final selection of studies to ensure accuracy and consistency. Data extracted from each study included study identification, methodological design, population characteristics, details of interventions and comparators, outcome measures, and numerical results. Outcome data reported in non-standard units were converted to standardized formats, and standard deviation (SD) was calculated from the standard error of the mean (SEM) when SEM was the only value provided.

### 2.4. Quality Assessment

Methodological quality was assessed using the Cochrane Risk of Bias in Non-Randomized Studies of Interventions (ROBINS-I) tool. Two reviewers (S.S. and J.S.) independently evaluated potential bias across seven domains: confounding, selection of participants, classification of interventions, deviations from intended interventions, missing data, measurement of outcomes, and selection of the reported result. Discrepancies between reviewers were resolved through discussion and consensus to ensure a consistent and accurate assessment. Detailed per-study, per-domain ROBINS-I judgments with textual justifications are presented in Supplementary Materials, Tables S6–S11.

### 2.5. Statistical Analysis

Statistical analyses were performed using Review Manager version 5.4.1 (Cochrane Collaboration, Copenhagen, Denmark). A prespecified random-effects model was selected to account for clinical and methodological variability across studies, including differences in design, populations, treatment protocols, and follow-up durations, incorporating both intra- and inter-study heterogeneity [17]. For continuous outcomes, such as survival time in days, pooled mean differences were calculated using the restricted maximum likelihood method, which provides unbiased estimates of between-study variance (τ^2^) even with a limited number of studies. The Hartung–Knapp–Sidik–Jonkman (HKSJ) method was applied to calculate 95% confidence intervals (CIs) to reduce type I error in small sample sizes [18,19,20]. For binary outcomes, such as survival rates at 6, 12, 24, and 36 months, data were logit-transformed to stabilize variance before pooling under a random-effects model. The DerSimonian–Laird method was used to estimate τ^2^, and the HKSJ method generated CIs to improve statistical accuracy in datasets with limited studies and high heterogeneity [20,21,22]. Median and range values reported in primary studies were converted to approximate means and standard deviations under a log-normal assumption using a hybrid algorithm refinement method [23,24]. Heterogeneity across studies was assessed using the I^2^ statistic, with thresholds of 25%, 50%, and 75% representing low, moderate, and high heterogeneity, respectively.

### 2.6. Sensitivity and Cross-Framework Analyses

Sensitivity analyses were performed to assess the robustness of the findings. These included (i) leave-one-out influence diagnostics for mean survival time, (ii) exclusions based on follow-up time and risk of missingness, and (iii) a cross-framework comparison contrasting pooled mean survival time with fixed-interval survival rates at 6, 12, 24, and 36 months. Survival rates were extracted from Kaplan–Meier (KM) where available and synthesized as risk differences using random-effects models. Formal statistical tests for funnel plot asymmetry such as Egger’s regression were not performed because fewer than ten studies were included and such tests are underpowered in small meta-analyses.

## 3. Results

### 3.1. Study Selection

**Figure 1:**
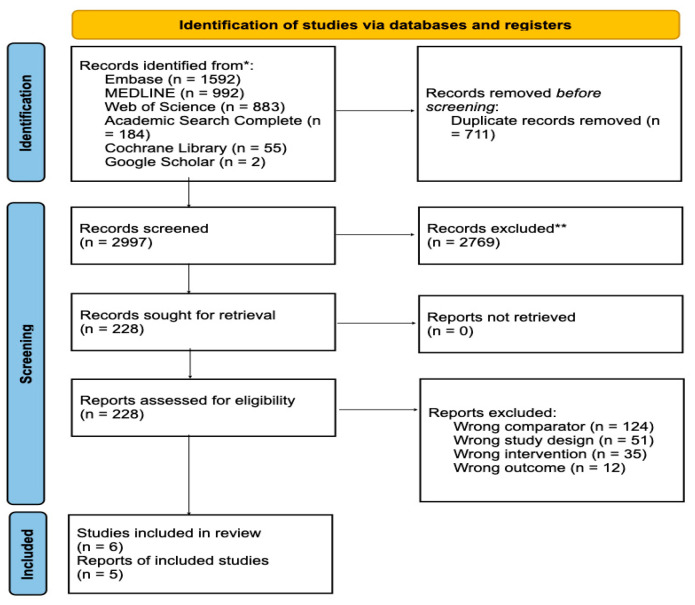
PRISMA 2020 flow diagram of the study selection process.

The comprehensive literature search identified a total of 3708 records: 3706 from five major electronic databases: Embase (1974–present via Ovid; n = 1592), MEDLINE (1946–present via Ovid; n = 992), Web of Science—All Databases (Clarivate Analytics; n = 883), the Cochrane Library (Wiley Online Library; n = 55), and Academic Search Complete (EBSCO; n = 184). An additional 2 records retrieved from screening the first 50 pages of Google Scholar, as the most relevant studies are typically captured in the initial pages. The study selection process is summarized in Figure 1. After removing 711 duplicates, 2997 titles and abstracts were screened. During the title and abstract screening, irrelevant studies were excluded, including those unrelated to CS in dogs or assessing non-pharmacological interventions such as dietary or behavioral management. This screening resulted in 228 articles being considered for full-text review. During the full-text review, 222 studies were excluded for multiple reasons: some investigated inappropriate comparators, such as surgical or radiotherapeutic interventions rather than medical treatments; others used study designs that did not meet methodological standards, including single case reports, narrative reviews, or uncontrolled observational studies; several evaluated pharmacological agents outside the scope of this review; and some did not report survival outcomes, focusing instead on biochemical or clinical endpoints. Following this process, 6 studies met all eligibility criteria and were included in the final systematic review, with 5 studies incorporated into the meta-analysis.

### 3.2. Study Characteristics

Five studies met the inclusion criteria and included naturally occurring CS in 295 dogs. The study characteristics are summarized in Table 1. Eligible articles assessed at least one pharmacological agent: trilostane, mitotane, ketoconazole, cabergoline, selegiline, or aminoglutethimide, and reported survival outcomes. Four studies supported a direct comparison between trilostane and mitotane. Study designs included randomized controlled trials and cohort studies, with variation in treatment dosage and examination. Most subjects were adult dogs, some with comorbid conditions. Survival outcomes were reported either as mean survival time in days or as survival rates at defined time points, with follow-up intervals from 6 to 36 months.

### 3.3. Risk of Bias

**Figure 2:**
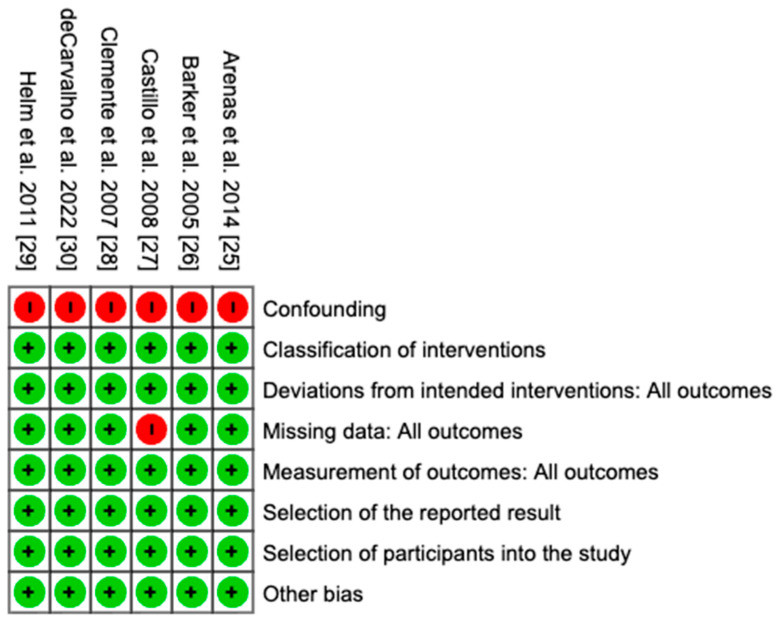
Cochrane risk of bias summary. Green circles indicate low risk, and red circles indicate high risk of bias across eight domains [25,26,27,28,29,30].

The risk of bias for the included studies was assessed using the Cochrane ROBINS-I tool, with results summarized in Figure 2. Most domain, including classification of interventions, deviations from intended interventions, measurement of outcomes, selection of the reported result, selection of participants into the study, and other sources of bias, were rated as low risk across all studies. The primary limitation identified was confounding, which received a high-risk rating for every study due to the non-randomized designs and the lack of control for important factors such as comorbidities and differences in treatment regimens. One study also received a high-risk rating for the missing data domain because survival information was incomplete for cases lost to follow-up. Overall, these assessments indicate that the studies generally maintained a low risk in most methodological domains.

### 3.4. Survival Outcomes

**Figure 3:**
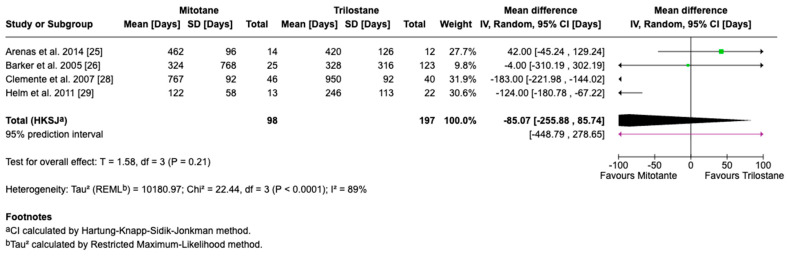
Forest plot comparing mean survival time (days) in dogs treated with mitotane versus trilostane across four studies [25,26,28,29].

**Figure 4:**
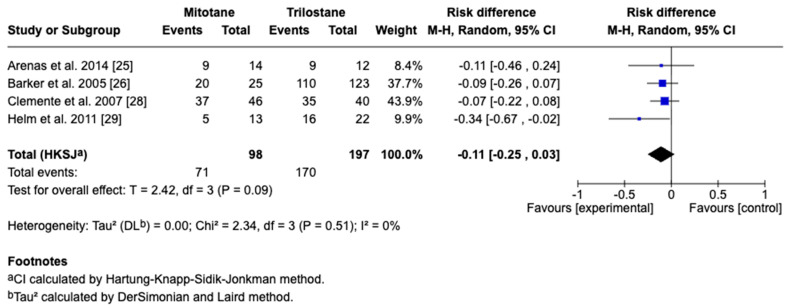
Forest plot comparing 6-month survival rates in dogs treated with mitotane versus trilostane across four studies [25,26,28,29].

**Figure 5:**
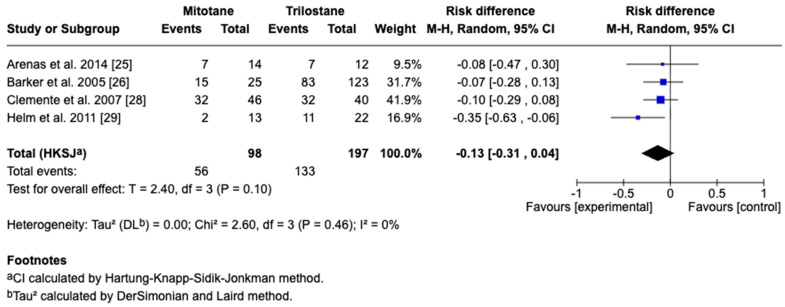
Forest plot comparing 12-month survival rates in dogs treated with mitotane versus trilostane across four studies [25,26,28,29].

**Figure 6:**
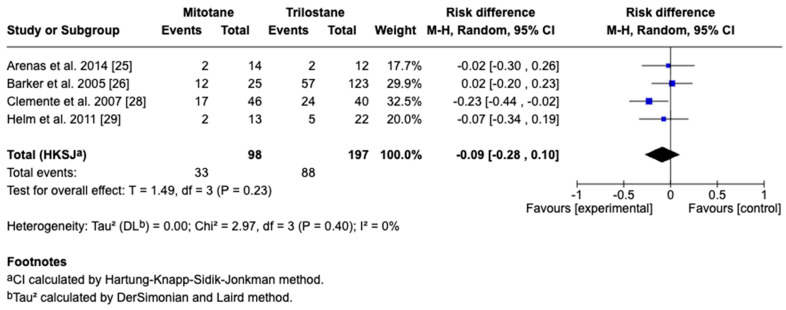
Forest plot comparing 24-month survival rates in dogs treated with mitotane versus trilostane across four studies [25,26,28,29].

**Figure 7:**
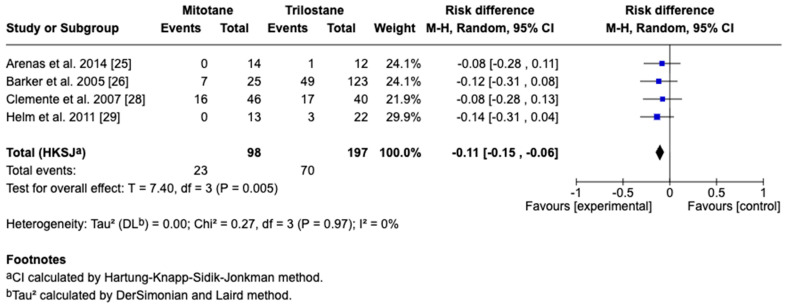
Forest plot comparing 36-month survival rates in dogs treated with mitotane versus trilostane across four studies [25,26,28,29].

The primary outcome of this study was survival time, reported in days as the mean difference with corresponding 95% CIs. For studies reporting medians and ranges, means and standard deviations were approximated using log-normal transformation methods for the inclusion of data in the meta-analysis. A random-effects model accounted for inter-study variability, with heterogeneity assessed by the I^2^ statistic, where 25%, 50%, and 75% represent low, moderate, and high heterogeneity, respectively. Five studies including 295 dogs met the inclusion criteria, with four providing direct comparisons between trilostane and mitotane. Results of the meta-analysis are presented in Figure 3, Figure 4, Figure 5, Figure 6 and Figure 7. The pooled analysis of survival time (Figure 3) revealed a mean difference of −85.1 days (95% CI: −255.9 to 85.7; *p* = 0.21), with substantial heterogeneity (I^2^ = 89%), indicating no statistically significant difference between treatments. In contrast, pooled analysis of survival rates at fixed intervals (Figure 4, Figure 5, Figure 6 and Figure 7) generally favored trilostane, showing an 11% higher survival at 6 months (*p* = 0.09), 13% at 12 months (*p* = 0.10), 9% at 24 months (*p* = 0.23), and a statistically significant 11% increase at 36 months (*p* = 0.005). The first three time points did not reach statistical significance, and no heterogeneity was detected at any of these intervals (I^2^ = 0%), respectively.

### 3.5. Sensitivity and Cross-Framework Comparison

Leave-one-out analyses for the primary outcome, mean survival time, indicated that no individual study altered the direction or significance of the pooled estimate (range of mean differences: –152 to –40 days; all *p* > 0.05 except one), and substantial heterogeneity persisted across models (I^2^ = 50–93%) despite exclusion of individual studies. Consistency across survival outcome types was assessed by comparing pooled mean differences in survival time with pooled fixed-interval survival rates. The mean difference favor trilostane (–85 days; 95% CI: −256 to +86; *p* = 0.21), with high heterogeneity (I^2^ = 89%), whereas survival rates at 6, 12, and 24 months showed no significant differences. At 36 months, survival was significantly higher in the trilostane group (risk difference −0.11; 95% CI: −0.15 to −0.06; *p* = 0.005), with no detectable heterogeneity (I^2^ = 0%), which indicates a consistent directional trend at longer follow-up despite differences in survival summary measures.

### 3.6. Subgroup Analysis

The review protocol outlined subgroup analyses to examine the potential impacts of drug dosage regimens, tumor etiology in CS dogs, and comorbidities on survival outcomes, as these factors are likely to influence treatment outcomes. However, none of the five included studies reported survival data stratified by these variables, and the individual animal-level data required for such analyses were not available. Subgroup meta-analyses based on fewer than two studies were excluded due to prevent unreliable or misleading interpretation due to insufficient statistical power. As a result, subgroup analyses were not performed. Instead, these factors were examined qualitatively to provide additional context and to support a more comprehensive interpretation of the available evidence.

## 4. Discussion

Cushing’s syndrome is a common endocrine disorder in dogs, primarily affecting middle-aged to older-aged animals and is reported across a broad range of breeds, with some studies noting an overrepresentation of certain small and medium-sized breeds [2,31,32]. No consistent sex predisposition has been identified [31,32,33,34]. Medical treatment is typically the first-line treatment, and among the studies included in this review, six pharmacological agents were reported: trilostane, mitotane, ketoconazole, cabergoline, selegiline, and aminoglutethimide. Among these, only trilostane and mitotane were evaluated in controlled studies reporting survival outcomes suitable for meta-analysis. Although both drugs remain widely used, they differ in mechanism of action, safety profiles, and clinical application.

Trilostane is a reversible, competitive inhibitor of 3β-hydroxysteroid dehydrogenase, an enzyme essential for the synthesis of cortisol, aldosterone, and sex hormones in the adrenal cortex [35]. This competitive inhibition lowers cortisol levels without causing long-term irreversible damage to adrenal tissue, allowing clinicians to adjust treatment protocols based on clinical signs and laboratory parameters, including serum cortisol and electrolyte measurements [35]. The drug’s reversible action and favorable safety profile make trilostane well-suited for long-term management, particularly in dogs with comorbidities such as diabetes mellitus, renal insufficiency, or cardiovascular disease, where careful monitoring is critical to minimize adverse effects [36]. Mitotane, in contrast, causes irreversible cytotoxic injury to the adrenal cortex, by specifically targeting cells within the zona fasciculata and zona reticularis [25,37]. This action effectively reduces cortisol production but carries a high risk of iatrogenic hypoadrenocorticism and adrenal insufficiency, which can result in life-threatening complications without appropriate intervention [29]. For this reason, mitotane is typically considered a second-line treatment, reserved for situations where trilostane is ineffective in disease management.

Other pharmacological agents, including ketoconazole, cabergoline, selegiline, and aminoglutethimide, have been evaluated primarily in small observational studies that lacked controlled survival data, limiting their use as primary treatments and confining them to refractory incidents of disease. Ketoconazole, an antifungal that inhibits cytochrome P450–dependent enzymes, can reduce cortisol synthesis but shows variable efficacy and carries a risk of hepatotoxicity, restricting its clinical application [38,39]. Cabergoline, a dopamine agonist acting on pituitary D2 receptors, has been used in pituitary-dependent hypercortisolism to suppress ACTH secretion, though responses are inconsistent and resistance often develops [40]. Selegiline, a monoamine oxidase B inhibitor that increases dopamine concentrations to inhibit ACTH release, has not demonstrated reliable clinical benefits and is currently rarely recommended [41,42]. Finally, aminoglutethimide, which blocks the conversion of cholesterol to pregnenolone and thereby reduces cortisol production, is limited by low efficacy and clinically undesirable effects such as sedation and ataxia [43,44]. Taken together, the evidence supports trilostane as the preferred first-line treatment, with mitotane as an alternative in resistant cases, and these other agents as adjunctive treatment for select patients.

The review identified a trend toward improved survival in dogs treated with trilostane compared to those receiving mitotane, particularly at extended follow-up intervals. Although the difference in average survival times between the two groups did not reach statistical significance, this finding likely reflects variability in sample sizes and heterogeneity among the included studies. Notably, the proportion of dogs surviving at 36 months was higher in the trilostane group, with an absolute difference of 11%. Survival rates at earlier intervals (6, 12, and 24 months) also tended to favor trilostane treatment, although these differences were not statistically significant. These data suggest that the benefits of trilostane may be cumulative over time, supporting its use as an effective long-term treatment. The reversible, non-cytotoxic mechanism of trilostane further reinforces its role as the preferred first-line treatment for CS in dogs, whereas mitotane remains an alternative for cases requiring a secondary treatment.

Several limitations should be considered when these findings are interpreted. First, the small number of eligible studies (n = 5) limits generalizability, even though meta-analyses can be conducted with as few as two studies [17,45,46]. None of the studies reported outcomes by disease subtype, such as pituitary-dependent versus adrenal-dependent hypercortisolism, despite important biological differences that may influence treatment response. Second, substantial clinical heterogeneity may have contributed to variable survival outcomes. Differences in trilostane dosing, such as once- versus twice-daily administration, use of mitotane induction versus maintenance protocols, and post-ACTH cortisol monitoring targets may have affected both efficacy and tolerability. The drugs had distinct mechanisms, with trilostane as a reversible inhibitor of cortisol synthesis and mitotane as an agent that causes irreversible cytotoxic injury to adrenal cortical cells, which may also have influenced long-term survival through disease control or treatment-related adverse events [35,36,37,38].

Third, methodological limitations further constrain interpretation. Time-to-event outcomes are ideally analyzed with hazard ratios (HRs) that account for censoring and variable follow-up, but HRs were rarely reported, and KM curves did not provide sufficient detail for reconstruction [47,48]. As a result, survival outcomes were synthesized with pooled mean differences and fixed-interval rates, with log-normal transformations applied where necessary, and the HKSJ method applied to reduce bias from small sample sizes and heterogeneity [22,29,49,50,51]. Sensitivity analyses, which included leave-one-out diagnostics, study-level exclusions, and cross-framework comparisons between pooled mean survival times and fixed-interval survival rates at 6, 12, 24, and 36 months, supported the robustness of the findings [52,53]. The 36-month survival rate significantly favored trilostane, which reinforced the mean survival trend despite limitations in time-to-event modeling. Finally, all included studies were non-randomized cohorts, which introduced potential confounding, limited control over treatment allocation, and reduced confidence in causal inference.

## 5. Conclusions

This systematic review and meta-analysis suggest that trilostane may provide superior long-term survival compared with mitotane in dogs with naturally occurring CS, with the survival benefit most evident at 36 months post-treatment. Trilostane acts through reversible inhibition of cortisol synthesis and has a comparatively favorable safety profile, which contributes to improved tolerability and suitability for prolonged treatment. This drug mechanism and safety profile allows clinicians to adjust treatment regimens based on clinical findings and laboratory results. Other pharmacological agents, including ketoconazole, cabergoline, selegiline, and aminoglutethimide, have also been evaluated; however, current evidence provides insufficient data about their effects on survival or practical use in clinical practice. The limitations surrounding these drugs reflect several factors, including the small number of studies, lack of disease subtype stratification, heterogeneity in study populations and treatment protocols, and reliance on non-randomized cohort designs with limited reporting of time-to-event outcomes. Collectively, these findings support the use of trilostane as the preferred first-line treatment in clinical practice, with mitotane reserved for cases resistant to trilostane, while the other agents serve as adjunctive treatments. From the information provided, well-designed prospective clinical trials should be considered for future studies. These studies should ideally include standardization protocols, specific disease subtype stratification, and detailed survival outcomes. As such, these studies could improve the information needed for guiding the long-term management of CS in dogs.

## Figures and Tables

**Table 1:** Key characteristics of included studies and the data extracted for analysis.

Author	Period	Study	Type	n	Age	Breed	Gender	Dose	Duration
Arenas et al., 2014 [25]	1994–2009	Retrospective	ADH	26	Mean 10.9 y (range 7–16)	13 breeds various represented	10 M 16 F	Mitotane: 50–75 mg/kg/day (9–30 days induction), then 75–100 mg/kg/week. Trilostane: 3 mg/kg PO q12.	Mitotane:15.4 months (range 2.0–37.0 months) Trilostane: 17.7 months (range 3.3–55.0 months)
Barker et al., 2005 [26]	1998–2003	Retrospective	PDH	148	Mean 9.6 ± 2.3 y (range 3.5–15.2)	44 breeds; toy (26%), terrier (22%) over-represented vs. UK registrations	75 M (45 intact, 30 neutered), 73 F (20 intact, 53 spayed)	Mitotane: Induction: 50 mg/kg PO q24 until clinical signs improved and post—ACTH cortisol < 120 nmol/L (4.32 µg/dL) Trilostane: No induction. 5–20 kg PO q24 = 60 mg 21–40 kg = 120 mg > 40 kg = 120–240 mg. Target: 4-h post-medication post-ACTH cortisol 40–120 nmol/L (1.44–4.32 µg/dL)	Mitotane: Continuous weekly maintenance after induction; continued lifelong or until death (median survival 708 d) Trilostane: Continuous once-daily (occasionally increased frequency) therapy lifelong or until death (median survival 662 d)
Castillo et al., 2008 [27]	NR	Prospective	PDH	63	Mean 9 y (range 3–14)	63 breeds; 40% mixed breed; the remainder were pure breeds	24 M 39 F	Ketoconazole: 20 mg kg^−1^ day^−1^ PO q24 Cabergoline: 0.07 mg kg^−1^ per week PO q48	Ketoconazole: Follow-up reported to 1 year for endocrine and MRI outcomes; survival followed to 4 years Cabergoline: Same: outcomes assessed at 1 year; responders followed up to 4 years.
Clemente et al., 2007 [28]	1994–2006	Retrospective	PDH	86	Mean 9.5 ± 2.0 y (range 6–13)	NR	25 M (3 neutered), 21 F (6 spayed)	Mitotane: 75–100 mg/kg/day (25-day induction), followed by lifelong glucocorticoid and mineralocorticoid replacement Trilostane: 3 mg/kg PO q12	Mitotane: 25-day induction with mitotane, followed by lifelong hormone-replacement therapy. Dogs were followed for six months until death (median survival 720 d) Trilostane: Treatment life-long; dose adjusted at re-checks based on clinical signs and post-ACTH cortisol. (median survival 900 d)
Helm et al., 2011 [29]	1996–2008	Retrospective	ADH	37	Mean 11 y (range 7–14)	14 breeds; 8 labradors, 7 crossbreeds, others ≤ 4	13 M 24 F (Neutered = 24/37)	Mitotane: Induction: 50 mg/kg PO q24 until clinical signs improved and post—ACTH cortisol < 120 nmol/L (4.32 µg/dL). Trilostane: No induction. PO q24	Mitotane: Continuous medical therapy after induction; weekly maintenance continued lifelong Trilostane: PO q24 (occasionally q12) therapy lifelong; dose adjusted

ACTH, adrenocorticotropic hormone; ADH, adrenal-dependent hypercortisolism; BW, body weight; d, days; F, female; kg, kilogram; M, male; mg, milligram; MRI, magnetic resonance imaging; n, number of animals; nmol/L, nanomoles per liter; NR, not reported; PDH, pituitary-dependent hypercortisolism; PO, per os (oral); q12, twice daily; q24, once daily; q48, every other day; µg/dL, micrograms per deciliter; y, years.

## Data Availability

Data are contained within the article and Supplementary Materials.

## Supplementary Materials

The following supporting information can be downloaded at: https://www.mdpi.com/article/10.3390/ani15202954/s1. Tables S1–S5 presents the search strategies for each database, and Tables S6–S11 presents the ROBINS-I risk of bias assessments of reach included article.

## Abbreviations

ACTH	Adrenocorticotropic hormone
ADH	Adrenal-dependent hypercortisolism
BW	Body weight
CI	Confidence interval
CS	Cushing’s syndrome
d	Days
F	Female
HKSJ	Hartung–Knapp–Sidik–Jonkman
kg	Kilogram
M	Male
mg	Milligram
MRI	Magnetic resonance imaging
n	Number of animals
nmol/L	Nanomoles per liter
NR	Not reported
PHD	Pituitary-dependent hypercortisolism
PO	Per os (oral)
PRIMSA	Preferred Reporting Items for Systematic Reviews and Meta-Analyses
q12	Twice daily
q24	Once daily
q48	Every other day
ROBINS-I	Risk of Bais in Non-Randomized Studies of Interventions
SD	Standard deviation
SEM	Standard error of the mean
τ^2^	Between-study variance
µg/dL	Micrograms per deciliter
y	years
