# Supplementary material for: Effectiveness of Medical Treatment on Survivability in Canine Cushing’s Syndrome: A Systematic Review and Meta-Analysis
Source: Animals (Basel). 2025 Oct 12;15(20):2954. doi: 10.3390/ani15202954 (PMC12560878; doi:10.3390/ani15202954)
Supplement: Supplementary file 1 [file animals-15-02954-s001.zip › Table_S1-S5_Search_Strategies.pdf]

Table S1. MEDLINE (1946–January 3, 2025) search strategy for medical treatments in canine Cushing's syndrome.

#	Search Statement	Results
1	exp Dogs/	349592
2	(canine* or dog or dogs or doggy or puppy or puppies or mongrel* or hound or hounds or pooch* or mutt or mutts or bitch*).mp.	411141
3	(terrier* or spaniel* or retriever* or mastiff* or pinscher* or collie* or poodle* or dachshund* or corgi* or shepherd* or sheepdog* or beagle* or coonhound* or bloodhound* or borzoi* or english foxhound* or greyhound* or harrier* or irish wolfhound* or otterhound* or rhodesian ridgeback* or scottish deerhound*).mp. [mp=title, book title, abstract, original title, name of substance word, subject heading word, floating sub-heading word, keyword heading word, organism supplementary concept word, protocol supplementary concept word, rare disease supplementary concept word, unique identifier, synonyms, population supplementary concept word, anatomy supplementary concept word]	30304
4	1 or 2 or 3	416323
5	exp Cushing Syndrome/ or exp Pituitary ACTH Hypersecretion/ or exp ACTH-Secreting Pituitary Adenoma/ or exp Adrenal Gland Neoplasms/ or exp Adrenocorticotropic Hormone/ or exp Adrenocortical Hyperfunction/ or exp Hydrocortisone/	163417
6	(cushing* or adrenocorticotropic hormone* or ACTH* or ((corticotrop* or pituitary or adrenal) adj2 (adenoma* or tumo?r*)) or hyperadrenocortic* or hypercortisol* or hydrocortisone*).mp.	176829
7	5 or 6	207477
8	exp Drug Therapy/ or exp Mitotane/ or exp Selegiline/ or exp Ketoconazole/ or exp Cabergoline/ or exp Aminoglutethimide/	1553745
9	(pharmacotherap* or mitotan* or "o,p-DDD" or lysodren or selegilin* or l-deprenyl or anipryl or eldepryl or carbex or zelepar or zelapar or trilostan* or vetoryl or desopan or modrastan* or modrenal or ketoconazol* or "R 41400" or "R41400" or "R41 40" or nizoral or cabergolin* or galastop or "FCE 21336" or caberlin* or cabaser or cabaseril or dostinex or aminoglutethimid* or elipten or cytadren or orimeten).mp.	68293
10	((medic* or pharmacolog* or drug*) adj2 (treat* or therap* or management* or intervention* or efficac* or effectiv* or safety or trial* or procedure*)).mp.	3241783
11	8 or 9 or 10	3997464
12	4 and 7 and 11	992

Table S2. Embase (1974–January 3, 2025) search strategy for medical treatments in canine Cushing's syndrome.

#	Search Statement	Results
1	exp Dogs/	298918
2	(canine* or dog or dogs or doggy or puppy or puppies or mongrel* or hound or hounds or pooch* or mutt or mutts or bitch*).mp.	382487
3	(terrier* or spaniel* or retriever* or mastiff* or pinscher* or collie* or poodle* or dachshund* or corgi* or shepherd* or sheepdog* or beagle* or coonhound* or bloodhound* or borzoi* or english foxhound* or greyhound* or harrier* or irish wolfhound* or otterhound* or rhodesian ridgeback* or scottish deerhound*).mp. [mp=title, abstract, heading word, drug trade name, original title, device manufacturer, drug manufacturer, device trade name, keyword heading word, floating subheading word, candidate term word]	41528
4	1 or 2 or 3	393169
5	exp cushing syndrome/ or exp cushing's disease/ or exp ACTH secreting adenoma/ or exp adrenal tumor/ or exp corticotropin/ or exp adrenal cortex hyperfunction/ or exp hypercortisolism/	128507
6	(cushing* or adrenocorticotropic hormone* or ACTH* or ((corticotrop* or pituitary or adrenal) adj2 (adenoma* or tumo?r*)) or hyperadrenocortic* or hypercortisol* or hydrocortisone*).mp.	260904
7	5 or 6	299417
8	exp drug therapy/ or exp mitotane/ or exp selegiline/ or exp trilostane/ or exp ketoconazole/ or exp cabergoline/ or exp aminoglutethimide/	3953420
9	(pharmacotherap* or mitotan* or "o,p-DDD" or lysodren or selegilin* or l-deprenyl or anipryl or eldepryl or carbex or zelepar or zelapar or trilostan* or vetoryl or desopan or modrastan* or modrenal or ketoconazol* or "R 41400" or "R41400" or "R41 40" or nizoral or cabergolin* or galastop or "FCE 21336" or caberlin* or cabaser or cabaseril or dostinex or aminoglutethimid* or elipten or cytadren or orimeten).mp.	135297
10	((medic* or pharmacolog* or drug*) adj2 (treat* or therap* or management* or intervention* or efficac* or effectiv* or safety or trial* or procedure*)).mp.	6746601
11	8 or 9 or 10	7917436
12	4 and 7 and 11	1592

Table S3. Web of Science (1955–January 3, 2025) search strategy for medical treatments in canine Cushing's syndrome.

#	Search Statement	Results
1	canine* or dog or dogs or doggy or puppy or puppies or mongrel* or hound or hounds or pooch* or mutt or mutts or bitch*	1027259
2	terrier* or spaniel* or retriever* or mastiff* or pinscher* or collie* or poodle* or dachshund* or corgi* or shepherd* or sheepdog* or beagle* or coonhound* or bloodhound* or borzoi* or "english foxhound*" or greyhound* or harrier* or "irish wolfhound*" or otterhound* or "rhodesian ridgeback*" or "scottish deerhound*"	102359
3	1 or 2	266533
4	(cushing* or "adrenocorticotropic hormone*" or ACTH* or ((corticotrop* or pituitary or adrenal) NEAR/2 (adenoma* or tumo?r*)) or hyperadrenocortic* or hypercortisol* or hydrocortisone*)	925065
5	(pharmacotherap* or mitotan* or "o,p-DDD" or lysodren or selegilin* or l-deprenyl or anipryl or eldepryl or carbex or zelepar or zelapar or trilostan* or vetoryl or desopan or modrastan* or modrenal or ketoconazol* or "R 41400" or "R41400" or "R41 40" or nizoral or cabergolin* or galastop or "FCE 21336" or caberlin* or cabaser or cabaseril or dostinex or aminoglutethimid* or elipten or cytadren or orimeten)	5790122
6	((medic* or pharmacolog* or drug*) NEAR/2 (treat* or therap* or management* or intervention* or efficac* or effectiv* or safety or trial* or procedure*))	1066124
7	5 or 6	6535171
8	3 and 4 and 7 (Exclude Medline)	883

Table S4. Academic Search Complete (1998–January 3, 2025) search strategy for medical treatments in canine Cushing’s syndrome.

#	Search Statement	Results
1	canine* or dog or dogs or doggy or puppy or puppies or mongrel* or hound or hounds or pooch* or mutt or mutts or bitch*	165908
2	terrier* or spaniel* or retriever* or mastiff* or pinscher* or collie* or poodle* or dachshund* or corgi* or shepherd* or sheepdog* or beagle* or coonhound* or bloodhound* or borzoi* or “english foxhound*” or greyhound* or harrier* or “irish wolfhound*” or otterhound* or “rhodesian ridgeback*” or “scottish deerhound*”	56265
3	1 or 2	207689
4	(cushing* or “adrenocorticotropic hormone*” or ACTH* or ((corticotrop* or pituitary or adrenal) N2 (adenoma* or tumo?r*)) or hyperadrenocortic* or hypercortisol* or hydrocortisone*)	43284
5	(pharmacotherap* or mitotan* or "o,p-DDD" or lysodren or selegilin* or l-deprenyl or anipryl or eldepryl or carbex or zelepar or zelapar or trilostan* or vetoryl or desopan or modrastan* or modrenal or ketoconazol* or "R 41400" or "R41400" or "R41 40" or nizoral or cabergolin* or galastop or "FCE 21336" or caberlin* or cabaser or cabaseril or dostinex or aminoglutethimid* or elipten or cytadren or orimeten)	61641
6	((medic* or pharmacolog* or drug*) N2 (treat* or therap* or management* or intervention* or efficac* or effectiv* or safety or trial* or procedure*))	699779
7	5 or 6	743258
8	3 and 4 and 7	184

Table S5. Cochrane Library (up to January 3, 2025) search strategy for medical treatments in canine Cushing's syndrome.

#	Search Statement	Results
1	MeSH descriptor: [Dogs] explode all trees	1149
2	canine* or dog or dogs or doggy or puppy or puppies or mongrel* or hound or hounds or pooch* or mutt or mutts or bitch*	5211
3	terrier* or spaniel* or retriever* or mastiff* or pinscher* or collie* or poodle* or dachshund* or corgi* or shepherd* or sheepdog* or beagle* or coonhound* or bloodhound* or borzoi* or (english NEXT foxhound*) or greyhound* or harrier* or (irish NEXT wolfhound*) or otterhound* or (rhodesian NEXT ridgeback*) or (scottish NEXT deerhound*)	3560
4	{or #1-#3}	8557
5	MeSH descriptor: [Cushing Syndrome] explode all trees	134
6	MeSH descriptor: [Pituitary ACTH Hypersecretion] explode all trees	50
7	MeSH descriptor: [ACTH-Secreting Pituitary Adenoma] explode all trees	28
8	MeSH descriptor: [Adrenal Gland Neoplasms] explode all trees	176
9	MeSH descriptor: [Adrenocorticotropic Hormone] explode all trees	1774
10	MeSH descriptor: [Adrenocortical Hyperfunction] explode all trees	290
11	MeSH descriptor: [Hydrocortisone] explode all trees	7799
12	(cushing* or (adrenocorticotropic NEXT hormone*) or ACTH* or ((corticotrop* or pituitary or adrenal) NEAR/2 (adenoma* or tumor* or tumour*)) or hyperadrenocortic* or hypercortisol* or hydrocortisone*)	14172
13	{or #5-#12}	14485
14	MeSH descriptor: [Drug Therapy] explode all trees	187818
15	MeSH descriptor: [Mitotane] explode all trees	21
16	MeSH descriptor: [Selegiline] explode all trees	292
17	MeSH descriptor: [Ketoconazole] explode all trees	667
18	MeSH descriptor: [Cabergoline] explode all trees	190
19	MeSH descriptor: [Aminoglutethimide] explode all trees	90
20	(pharmacotherap* or mitotan* or "o,p-DDD" or lysodren or selegilin* or l-deprenyl or anipryl or eldepryl or carbex or zelepar or zelapar or trilostan* or vetoryl or desopan or modrastan* or modrenal or ketoconazol* or "R 41400" or "R41400" or "R41 40" or nizoral or cabergolin* or galastop or "FCE 21336" or caberlin* or cabaser or cabaseril or dostinex or aminoglutethimid* or elipten or cytadren or orimeten)	15246
21	((medic* or pharmacolog* or drug*) NEAR/2 (treat* or therap* or management* or intervention* or efficac* or effectiv* or safety or trial* or procedure*))	640134
22	{or #14-#21}	689487
23	#4 AND #13 AND #22	55
