# Supplementary material for: Effectiveness of Medical Treatment on Survivability in Canine Cushing’s Syndrome: A Systematic Review and Meta-Analysis
Source: Animals (Basel). 2025 Oct 12;15(20):2954. doi: 10.3390/ani15202954 (PMC12560878; doi:10.3390/ani15202954)
Supplement: Supplementary file 1 [file animals-15-02954-s001.zip › Table_S6-S11_Risk_of_Bias_Assessments.pdf]

Table S6. Risk of bias assessment for Arenas et al. (2014) on survival in dogs with Cushing's syndrome.

**Bias Domain**		**Risk of Bias Judgement**
Bias due to confounding	Non-randomized, retrospective design comparing two treatments across different time periods. Baseline characteristics were reported as similar, but treatment selection was not randomized, introducing the risk of confounding.	Serious
Bias in classification of interventions	Medicla interventions (mitotane and trilostane) were applied using established protocols, but there were treatment differences and a lack of randomization that introduced classification inaccuracies. Additionally, two dogs underwent adrenalectomy after introducing trilostane, potentially complicating group classification.	Moderate
Bias in selection of participants into the study	Dogs were retrospectively included from two clinics over 15 years based on ADH diagnosis and treatment. The study does not specify whether any eligible cases were excluded after treatment was initiated, introducing potential selection bias.	Moderate
Bias due to deviations from intended interventions	Treatment protocols were consistent, with therapy adjustments based on clinical monitoring. No deviations were reported that would affect the outcomes.	Low
Bias due to missing data	Two dogs in the trilostane group were not followed up after adrenalectomy, and some causes of deaths were undetermined, contributing to a moderate missing data risk.	Moderate
Bias in measurement of outcomes	Outcomes were based on objective survival data and ACTH stimulation testing. Some clinical signs relied on self-reporting, introducing moderate measurement variability.	Moderate
Bias in selection of the reported results	The study focused on survival and predefined prognostic indicators. However, as a retrospective analysis with no registered protocol, it is unclear whether additional outcomes or alternative models were also assessed.	Moderate
Overall risk of bias	Serious overall risk due to non-randomized design, moderate missing data, and absence of protocol registration. Despite these limitations, the study provides comparative survival insights.	Serious

Table S7. Risk of bias assessment for Barker et al. (2005) on survival in dogs with Cushing's syndrome.

Bias Domain	Assessment Justification	Risk of Bias Judgement
Bias due to confounding	Retrospective design with variations in treatment protocols between referral centers. Although baseline characteristics were compared and appeared similar, there was no randomization of animal treatment, and treatment selection bias cannot be excluded.	Serious
Bias in classification of interventions	Treatment groups were based on clinical records, but center-specific treatment preferences potentially introduce treatment allocation bias. No misclassification of intervention after assignment was noted.	Moderate
Bias in selection of participants into the study	Participants were selected retrospectively from three referral hospitals. Dogs lacking sufficient medical records or dogs treated with more than one drug were excluded, which may have introduced bias linked to disease prognosis or data completeness.	Moderate
Bias due to deviations from intended interventions	Treatments followed established protocols without evidence of deviation from therapy. Adjustments to treatment (drug administered) were based on clinical response to treatment and test results, reflecting typical clinical practice.	Low
Bias due to missing data	Several cases lacked recorded causes for death or follow-up information post-treatment (28/82 dogs). This raises concerns about missing data, especially on outcomes associated with cause-specific mortality.	Moderate
Bias in measurement of outcomes	Survival was accurately recorded, but reporting of data for cause of death was inconsistent, limiting definitive conclusions on treatment-related mortality. Survival duration is less prone to measurement bias.	Low
Bias in selection of the reported results	Only survival was reported as the primary outcome, with no indication of secondary outcomes or protocol registration. The absence of a predefined analysis plan limits transparency and interpretation of the data and raises the possibility of selective reporting.	Moderate
Overall risk of bias	Cumulative risk is a serious limitation due to confounding effects, missing outcome data, and lack of protocol transparency. Nevertheless, the study's findings were used, given the large sample size.	Serious

Table S8. Risk of bias assessment for Clemente et al. (2007) on survival in dogs with Cushing's syndrome.

Bias Domain	Assessment Justification	Risk of Bias Judgement
Bias due to confounding	Retrospective cohort design with two treatment groups developed from various time periods (historical controls). Baseline characteristics were similar, but the non-random selection of dogs within the study was a potential source of bias.	Serious
Bias in classification of interventions	Medical interventions were well documented and based on clinical observation, with the application of standardized treatment protocols for mitotane and trilostane. However, the time periods of treatment were not aligned, raising potential historical bias.	Moderate
Bias in selection of participants into the study	Dogs diagnosed with PDH were retrospectively diagnosed based on treatment records. Four mitotane-treated dogs were excluded after intervention, raising concerns about post-treatment selection bias.	Moderate
Bias due to deviations from intended interventions	No deviations from treatment regimens were reported, and treatments were administered according to well-described protocols with adjustments based only on clinical observations.	Low
Bias due to missing data	Four dogs in the mitotane group were excluded due to loss to follow-up or change in treatment, introducing potential attrition bias. Comprehensive follow-up information varied between the study groups.	Moderate
Bias in measurement of outcomes	Objective clinical and laboratory measures (ACTH stimulation tests, survival data) were used, minimizing measurement bias. However, some outcomes, like clinical improvement during therapy, were based on owner reports.	Low
Bias in selection of the reported results	The study emphasized survival time and treatment that were associated with potential drug toxicity. Although reported well, the lack of protocol registration or consideration of other measured variables introduced the possibility of selective reporting.	Moderate
Overall risk of bias	Overall risk is serious due to non-randomized design, historical controls, exclusions, and absence of protocol transparency, though the data provide comparative insights on treatment outcomes.	Serious

Table S9. Risk of bias assessment for Helm et al. (2011) on survival in dogs with Cushing's syndrome.

Bias Domain	Assessment Justification	Risk of Bias Judgement
Bias due to confounding	Retrospective observational design without randomization introduces the risk of confounding. Statistical adjustment measurements were explored, but the small sample size and lack of controls for all possible confounders limit the study's reliability. No pre-specified list of confounders or advanced adjustment methods was employed in the study.	Serious
Bias in classification of interventions	Medical intervention assignment was based on treatment records, but the choice of therapy choice was influenced by drug availability and clinician preference, introducing potential selection bias. There is no indication of misclassification of treatment protocols once treatment to the individual animals was assigned.	Moderate
Bias due to deviations from intended interventions	No deviations from the intended medical interventions were reported. Treatments were delivered according to the protocols as described, and adherence to these protocols was consistent across study groups.	Low
Bias in selection of participants into the study	Dogs with ADH were retrospectively identified from four centers. Some information was excluded due to incomplete documentation of the dogs, and differences in diagnostic protocols may have affected the consistency of study inclusion criteria.	Moderate
Bias due to missing data	Some data was incomplete on the causes of death and staging of metastatic disease. However, survival of the dogs was recorded for all, except for one dog, minimizing the impact of missing data on the primary outcome. The retrospective nature of the study can limit the utility of the data in comparison to prospective studies.	Moderate
Bias in measurement of outcomes	Outcome was survival time, a defined measure, reducing the likelihood of measurement bias. However, reliance on medical records on the effectiveness of treatment could potentially introduce some minor variations in treatment efficacy.	Low
Bias in selection of the reported results	The study reported survival as the primary outcome. Within the study, there was no prespecified protocol, and given the multicenter design of the investigation, it remains uncertain whether other measured outcomes were omitted from the conclusion of the study.	Moderate
Overall risk of bias	The combination of serious confounding, moderate classification bias, and significant selective reporting risk results in a potential overall serious risk of bias. This can be expected for retrospective observational studies with small sample sizes and no protocol registration.	Serious

Table S10. Risk of bias assessment for Castillo et al. (2008) on survival in dogs with Cushing's syndrome.

Bias Domain	Assessment Justification	Risk of Bias Judgement
Bias due to confounding	Dogs were allocated in a non-randomized manner that employed parallel treatment cohorts, introducing selection bias. Baseline medical characteristics of the individual dogs were not well aligned or randomly selected into treatment groups.	Serious
Bias in classification of interventions	Medical interventions were accurately classified based on recorded treatments. However, the method for allocating every third dog to the control group potentially creates systematic assignment bias.	Moderate
Bias in selection of participants into the study	Dogs were grouped based on treatment response. Dogs that failed to respond to cabergoline were excluded from further analysis, introducing post-baseline selection bias.	Moderate
Bias due to deviations from intended interventions	Treatments were provided according to protocol without any reported treatment variation. Adjustments to therapy were based on clinical responses.	Low
Bias due to missing data	Dogs not responding to treatment within three months were removed from the primary analysis of the study, which may introduce bias by excluding non-responders, inaccurately elevating perceived treatment effectiveness.	Serious
Bias in measurement of outcomes	Objective measures such as Magnetic Resonance Imaging (MRI) and blood hormonal assays were well reported. Nevertheless, some outcomes, such as clinical improvement, relied solely on the owner's assessment of the dog's health, which may introduce subjective bias.	Moderate
Bias in selection of the reported results	Reported outcomes were included in treatment response, hormonal levels, MRI, and survival. The exclusion of non-responders and the absence of predefined protocol measurements raise concerns that only favorable results were highlighted in the study.	Moderate
Overall risk of bias	Overall, non-randomized allocation of dogs into treatment groups, exclusion of non-responders, and lack of detailed treatment protocols introduced a serious risk of bias.	Serious

Table S11. Risk of bias assessment for deCarvalho et al. (2022) on cortisol secretion in dogs with Cushing's syndrome.

Bias Domain	Assessment Justification	Risk of Bias Judgement
Bias due to confounding	The study was randomized but non-blinded, allowing for biased clinical evaluation and treatment outcomes. A small sample size (n=15) can induce significant variability. The randomization process for allocating treatment to CS dogs was not well described in details needed to rule out selection bias.	Serious
Bias in classification of interventions	Assignment of medical intervention was randomized, reducing classification bias. However, the lack of blinding and small group sizes can introduce an assessment intervention bias.	Moderate
Bias in selection of participants into the study	Dogs with confirmed PDH were randomized into treatment groups. Although inclusion was based on clear criteria, the small sample size and limited information on allocating dogs into treatment groups may affect accurate comparability between groups.	Moderate
Bias due to deviations from intended interventions	No deviations in the therapy regimens were reported. Both groups received described treatments with dosage adjustments based on clinical observations.	Low
Bias due to missing data	Three dogs either failed in follow-up assessment or died from unrelated causes prior to completion of the study, which introduces a moderate risk of animal attrition bias, particularly given the small sample size of the study.	Moderate
Bias in measurement of outcomes	Objective measures like ACTH, cortisol, and ultrasound were recorded, potentially reducing measurement bias. However, some clinical outcomes relied on owner-reported assessment of the dog's health or subjective clinical scoring, which can introduce notable measurement variability.	Moderate
Bias in selection of the reported results	Outcomes were reported as specified in the study (e.g., ACTH, adrenal thickness, clinical response), but the investigation, however, lacked protocol registration, and it is undetermined whether all intended endpoints were provided.	Moderate
Overall risk of bias	Overall risk is serious due to the small sample size, non-blinded assessment, and potential selective reporting of information. Despite these limitations, the randomized design provides some confidence in the accuracy and, as such, utility of primary outcomes.	Serious
